# Advancements in Pharmacotherapy for Noncancerous Manifestations of HPV

**DOI:** 10.3390/jcm4050832

**Published:** 2015-04-24

**Authors:** Ramya Kollipara, Erfon Ekhlassi, Christopher Downing, Jacqueline Guidry, Michael Lee, Stephen K. Tyring

**Affiliations:** 1 Center for Clinical Studies, Houston 77004, TX, USA; E-Mails: cdowning@ccstexas.com (C.D.); jguidry@ccstexas.com (J.G.); mlee@ccstexas.com (M.L.); styring@ccstexas.com (S.T.); 2 Department of Dermatology, the University of Texas Health Science Center at Houston, Houston 77030, TX, USA; E-Mail: Erfon.Ekhlassi@uth.tmc.edu

**Keywords:** pharmacotherapy, HPV, warts, condyloma, treatment

## Abstract

Human papillomavirus (HPV) is the most common sexually transmitted disease. Via infection of the basal epithelial cells, HPV causes numerous malignancies and noncancerous cutaneous manifestations. Noncancerous cutaneous manifestations of HPV, including common, plantar, plane, and anogenital warts, are among the most common reasons for an office visit. Although there are various therapies available, they are notoriously difficult to treat. HPV treatments can be grouped into destructive (cantharidin, salicylic acid), virucidal (cidofovir, interferon-α), antimitotic (bleomycin, podophyllotoxin, 5-fluorouracil), immunotherapy (*Candida* antigen, contact allergen immunotherapy, imiquimod) or miscellaneous (trichloroacetic acid, polyphenon E). The mechanism of action, recent efficacy data, safety profile and recommended regimen for each of these treatment modalities is discussed.

## 1. Introduction

Human papillomavirus (HPV), a double stranded DNA virus, is the most common sexually transmitted infection in the world. By infecting the basal cells of the epithelial mucosa, HPV causes malignancies (cervical, penile, vulvar, vaginal, anal and oropharyngeal) and noncancerous cutaneous manifestations [1]. Noncancerous cutaneous manifestations include common warts (verruca vulgaris), plantar warts, plane warts and anogenital warts [2].

Cutaneous and anogenital warts are common reasons for a dermatology office visit. Although there are various therapies available, they are extremely difficult to treat and none of the treatment modalities are curative. Therapy should be chosen based on lesion location, size, number, cost, adverse reactions associated with therapy, patient comorbidities and age. If three months of treatment does not yield improvement, a new therapy should be attempted [3]. HPV treatments can be grouped into destructive, virucidal, antimitotic, immunotherapy or miscellaneous therapy (Table 1).

**Table 1:** Efficacy and safety profile of HPV treatments.

Therapy (Dosing)	Regimen	Clearance	Adverse reactions	Indications
**Destructive**				
Cantharidin (0.7% or 3%)	Apply in office, cover for 2–12 h and then wash off; repeat every 2–4 weeks	80% with cantharidin 96% with cantharidin 1%, salicylic acid 30% and podophyllotoxin 5% compound for plantar warts	burning, erythema, pain, pruritus, donut warts	Plantar and periungual warts
Salicylic acid (15%–60%)	Solution and gel: 2–3 times daily Disc: apply and occlude for 48 h Wart should be filed, soaked in water for 5 min and dried before therapy. Maximum of 12 weeks	0–80%	irritation, flaking, peeling	Cutaneous warts, contraindicated in anogenital and facial warts due to severe irritation and potential scarring
**Virucidal**				
Cidofovir (Topical: 1%–2%, Intralesional: 15 mg/mL)	Topical: 1–2 times daily; maximum 10 weeks Intralesional: 1 injection per lesion per month	47% (topical) 99% (intralesional)	Topical: pain, pruritus, rash Intralesional: pain, burning sensation, itching, erythema, post-inflammatory hyperpigmentation	Anogenital, periungual, plantar
Interferon-α (10^6^ IU)	Intralesional: 2–3 times weekly; maximum 8 weeks	66%	pain, headache, mild-moderate flu-like symptoms	Genital
**Antimitotic**				
Bleomycin (dosing depends on technique employed)	Various techniques available	14%–99%	pain, burning, erythema, swelling, flagellate hyperpigmentation, lymphangitis, Raynaud’s phenomenon	Cutaneous
Podophyllotoxin (0.5%)	2 times day for 3 days, followed by a discontinuation of usage for 4 days; repeat this cycle every week or a maximum of 4 weeks	56%–72%	erythema, swelling, mild pain, erosion, pruritus	Anogenital
5-fluorouracil (Topical: 5%, Intralesional: 3 mg/mL)	Topical: 1–2 times daily for 6 weeks Intralesional: weekly; maximum of 6 weeks	71%	pain, irritation, local cutaneous reactions, progressive ulceration	Genital and periungual
**Immunotherapy**				
Candida antigen (0.3 mL)	0.3 mL injected intradermally into largest lesion every 3 weeks; maximum of 3 treatments	56%–78%	erythema, pain	Cutaneous
Imiquimod (2.5%, 3.75%, 5%)	overnight 3 times a week; maximum of 16 weeks	30%–70%	erythema, erosions, burning, pruritus, psoriasiform eruptions, mucosal ulcerations, hyperpigmentation	Anogenital
Contact allergen immunotherapy (dosing and administration explained in column to the right)	DPCP: apply 0.1% or 2% solution, occlude for 48 h; apply increasing strengths until eczematous reaction is seen; repeat weekly SADBE: apply 1 or 2% solution; apply increasing strengths every 2–4 weeks	85% (DPCP) 69% (SADBE) 80% (DNCB)	erythema, desquamation, cutaneous edema, pruritus, burning, pain, acute contact dermatitis, blisters, hypopigmentation	Periungual and cutaneous (DPCP and SADBE, respectively)
**Other**				
Polyphenon E (10%–15%)	3 times daily; maximum of 16 weeks	54%–65%	erythema, pruritus, burning, pain, erosion, ulceration, induration, vesiculation	External genital and perianal
Trichloroacetic acid (80%–90%)	Weekly; maximum 10 treatments	81%	local discomfort, ulceration, scab formation	Genital

Because this is a review of treatments, it is important to note the natural history of warts in order to determine the efficaciousness of therapy. In a study of 1000 children, overall incidence of warts increased from 18% to 25% in 2 years when treatment was withheld. Two-thirds of the original lesions spontaneously regressed within two years [4].

## 2. Destructive Therapy

### 2.1. Cantharidin

Cantharidin, a vesicant derived from blister beetles, has long been used for treatment of cutaneous warts. It is utilized in a formulation consisting of 0.7% cantharidin and either acetone, ether or alcohol. Application of cantharidin to the epidermis results in activation of proteases, which trigger dissolution of desmosomal junctions and acantholysis [5].

Since 1992, this drug is no longer usable in the US, but studies have shown a cure rate up to 80% in periungual and plantar warts [6]. Combination therapy of cantharidin 1%, salicylic acid 30%, and podophyllotoxin 5% with subsequent debridement has shown a 96% cure rate for plantar warts with most patients requiring only one treatment [7].

Cantharidin is to be applied only in the office as oral consumption can lead to death. After application, the lesion should be covered and the cantharidin should be washed off two to 12 h later. Blistering occurs 24 to 48 h after application. Longer application or occlusion time increases blistering [8]. The treated skin heals in 4 to 7 days [5]. Repeat application should occur every 2 to 4 weeks [9]. Cantharidin is contraindicated for the facial, genital and perianal regions as well as the mucosal membranes [5,9]. Furthermore, cantharidin use near the eyelids or eyes is discouraged due to the risk of scleral erosion [5]. If used properly, side effects are rare; however, the most common side effects associated with cantharidin are burning, erythema, pain and pruritus. Also, cantharidin increases the risk of developing donut warts, or warts where the central portion resolves but the peripheral portion persists [9]. With appropriate physician application, cantharidin typically does not lead to scarring [5].

### 2.2. Salicylic Acid

Salicylic acid is an over-the-counter treatment that causes shedding of HPV-infected tissue. It is generally used for thicker lesions such as plantar or periungual warts. It exists as a solution, gel, and discs immersed in solution. While the solution and gel are to be used 2 to 3 times daily, the discs are applied and occluded for 48 h after removal. Prior to application of salicylic acid, the wart should be pared down, soaked in water for five minutes and dried. The maximal duration of treatment is 12 weeks [3]. A 2012 meta-analysis found that the use of salicylic acid to treat cutaneous warts had mixed results compared to placebo, varying from 0 to 80%. Overall, salicylic acid was noted to have some moderate superiority in efficacy *versus* placebo [10]. The side effects of salicylic acid include irritation, flaking and peeling [3]. Use of salicylic acid is contraindicated in facial and anogenital warts due to severe irritation and scarring [11].

## 3. Virucidal Therapy

### 3.1. Cidofovir

Cidofovir is an acyclic nucleoside phosphonate that acts as an alternate substrate for DNA polymerase and thus competitively inhibits this enzyme. Cidofovir binds and inhibits both viral and cellular DNA polymerase, stopping replication. The mechanism of action of cidofovir in HPV infected cells is not fully understood, but it is believed that its ability to halt host cell replication machinery in proliferating cells leads to apoptosis, and virustatic control of HPV infection [12].

Although cidofovir is only FDA approved for CMV retinitis in human immunodeficiency virus (HIV) patients, there are many reports of successful topical and intralesional use for HPV lesions [13,14]. Intravenous cidofovir is avoided as it is associated with nephrotoxicity, ocular injury (anterior uveitis, retinal detachment, iritis and permanent loss of vision), and possible teratogenicity [15]. Although topical cidofovir has been used for warts on the oral mucosa, hands and anogenital region, most of the efficacy data has been collected based on the latter diagnosis [16]. In one study, 47% and 0 of patients had a complete response to topical 1% cidofovir and placebo respectively. Furthermore, 37% and 18% of patients had partial resolution with cidofovir and placebo respectively [17]. In children, off-label use of cidofovir has been used to control the growth of papillomas in recurrent respiratory papillomatosis (RRP), a benign, multi-focal neoplasm caused by HPV infection. Prospective studies have shown partial to complete resolution of mucosal papillomas [18]. Documented side effects of cidofovir in treating RRP include cutaneous rash, headache, local inflammatory response, vocal cord scarring, compromise of airway, disorders of hematologic and chemical parameters in blood [19]. Gilead, the makers of cidofovir, reported a ban on its off-label use of cidofovir in 2011 due to concern for its side effects.

The recommended topical cidofovir concentration is 1% to 3% applied 1 to 2 times daily. Treatment should be stopped if there is no response after 10 weeks [14]. The most common side effects of topical cidofovir are pain, pruritus and rash at the application site. There has been at least one case of renal failure reported in the literature [20]. In one study using intralesional cidofovir 15 mg/mL once a month, 276 of 280 patients had complete wart clearance. The most common adverse events with these injections were pain, burning sensation, itching, erythema, and post-inflammatory hyperpigmentation [15]. More studies are needed to further evaluate the efficacy of cidofovir.

### 3.2. Interferon-α

Interferons are low-molecular weight glycoproteins that have antiviral, antiproliferative, antitumoral and immunomodulatory effects [21,22]. Interferon therapy can be topical, intralesional or systemic; however, topical interferon is not efficacious as it does not penetrate the stratum corneum [9,17].

Intralesional and systemic interferon have shown efficacy in genital wart clearance, but are not first line given the associated side effects and exorbitant costs [17,23]. Intralesional interferon has demonstrated success rates up to 66% in clinical trials [24]. Local surgery and podophyllotoxin have been shown to increase the effectiveness of intralesional interferon [17]. One meta-analysis showed a recurrence rate of 21% with interferon *versus* 34% with placebo [25]. Side effects of intralesional interferon are pain, headache and mild-moderate flu-like symptoms [24]. In an initial study, systemic interferon therapy induced genital wart clearance, decreased viral load and increased CD4+ T cell count in immunocompromised HIV patients with recalcitrant anogenital warts [26]. Side effects of systemic interferon are fever, headache, myalgia, myelosuppression, cardiotoxicity (arrhythmias, dilated cardiomyopathy and ischemic heart disease) and graft rejection in transplant patients. The risk of graft rejection is feared with intralesional interferon as well [17].

## 4. Antimitotic Therapy

### 4.1. Bleomycin

Bleomycin, a glycopeptide antibiotic produced by *Streptomyces verticillus*, can be used to treat recalcitrant cutaneous warts. It binds to the guanosine-cytosine-rich areas of DNA, creating extremely unstable free radicals that cause single-strand breaks and ultimately apoptosis. Certain tissues in the body have increased sensitivity to bleomycin, such as the skin [27]. There is some debate on the effectiveness of intralesional bleomycin because of the varying data on cure rates, which range from 14% to 99% [28]. One study compared the efficacy of intralesional bleomycin to cryotherapy, with a statistical difference in clearance rates between the two. Intralesional bleomycin showed a 95 to 97% resolution rate *versus* a 76% to 82% clearance rate with cryotherapy [28].

Multiple bleomycin administration techniques have been studied. A bifurcated needle boasts both successful delivery and efficacy with one study achieving a cure rate of 92% after one treatment. This technique requires administering 0.001 units of bleomycin while puncturing the wart compared to the intralesional method which requires a 0.2 unit injection of bleomycin into the base of the wart [29]. Another study dropped 1 mg/ml bleomycin solution onto the wart and then “pricked” the lesion with a 28-gauge Monolet lancet needle. This technique had a 92% clearance rate [30].

Intralesional bleomycin is to be injected once every 3 weeks until clearance of the verruca or a maximum of four treatments, whichever occurs first [28]. Adverse effects include injection site pain, burning, erythema and swelling [11]. There have been cases of flagellate hyperpigmentation and lymphangitis [31,32]. Raynaud’s phenomenon with potential cutaneous toxicity and loss of fingernails has been seen as well [33,34]. Intralesional bleomycin should be avoided in pregnant or lactating women, infants and children, immunosuppressed patients and patients with collagen vascular disease [33].

### 4.2. Podophyllotoxin

Podophyllotoxin is a derivate of podophyllin, a resin found in the roots and rhizomes of *Podophyllum peltatum*. Podophyllotoxin is an antimitotic agent that interferes with viral replication by inhibiting tubulin polymerization, thus preventing spindle formation. This leads to arrest in metaphase and disruption of the cell cycle, triggering necrosis of HPV-infected host cells [35,36]. Podophyllotoxin is useful in treating condylomata acuminata [37].

One meta-analysis showed that 56% of patients treated with podophyllotoxin had complete resolution of anogenital warts [37]. A more recent study reported that 72% of patients had complete clearance of warts with podophyllotoxin [38]. Podophyllotoxin also has a high rate of relapse with 79% of patients in one study showing wart recurrence [39].

Podophyllotoxin is self-administered in a 0.5% concentrated cream or solution. It should be applied twice a day on the affected area for three consecutive days, followed by a discontinuation of usage for 4 days. This cycle is repeated again every week until resolution of warts or a maximum of 4 weeks [40]. Side effects are minimal and include local erythema, swelling, mild pain, erosion, and pruritus [37,41]. Although podophyllotoxin is easily used at home, it is relatively expensive compared to other therapies [17].

### 4.3. 5-Fluorouracil

5-Fluorouracil (5-FU) was originally developed as an antineoplastic metabolite, but has been used as an antiviral agent as well. It is an analogue of the nucleic acid uracil, and becomes a “decoy” molecule that inhibits thymidylate synthase, an enzyme necessary for thymidine synthesis. This leads to inhibition of DNA and RNA synthesis, which prevents cellular replication and proliferation [42,43].

A meta-analysis of three different studies showed that 5-FU was superior to placebo. Seventy one percent of patients treated with 5-FU had complete resolution of their anogenital warts *versus* 23% of patients treated with placebo. One of those studies reported a recurrence of lesions in one patient who originally had complete resolution [44]. 5-FU can be administered as a topical gel or by intralesional injection. The 5% topical formulation is to be self-applied one to two times a day for six weeks, while the intralesional injection of 5-FU is administered once a week for a maximum of six weeks at a concentration of 3 mg/mL [45,46].

Adverse effects of 5-FU appear minimal, including injection site pain and irritation, local cutaneous reactions, and progressive ulceration. Contraindications for the use of 5-fluorouracil are pregnancy and patients who are immunodeficient [46]. There is one reported case of melonychia while treating periungual warts with 5-FU [47].

## 5. Immunotherapy

### 5.1. Candida Antigen

Intradermal injection of *Candida* antigen upregulates the cell-mediated immune response, augmenting the overall clearance of the HPV virus [9]. Specifically, a recent study revealed two times the pretreatment levels of peripheral blood mononuclear cells after *Candida* antigen injection. The overall immunologic mechanism for this therapy is unclear, but its efficacy has been proven. Other antigens studied include mumps and trichophyton.

A 2013 efficacy study showed that 56% (19/34) of patients had complete resolution of warts at all places on the body, 6% (2/34) had partial or complete resolution of the treated wart with no effect on untreated lesions, and 38% (13/34) had no response after *Candida* injection [48]. Another study reported clearance of distant untreated warts in 78% of patients who had complete resolution of treated warts [49]. It is believed that this phenomenon is due to upregulation of the patient’s immune response, leading to distant lesion clearing.

Candida antigen should be used as first-line treatment in immunocompetent patients with multiple (>5) or large (>1 cm) warts. It may also be used as second-line treatment in patients who have failed cryotherapy. The recommended dosing regimen is 0.3 mL of Candida antigen injected intradermally into the largest lesion every three weeks until complete clearance of the wart or a maximum of three treatments. The injection should be administered as superficially as possible in order to achieve maximal delivery with minimal pain [49].

Side effects include mild erythema and pain at the site of injection. There is one reported case of vitiligo and another case of painful, purple discoloration at the site of injection [50,51,52].

### 5.2. Imiquimod

The FDA approved imiquimod in 1997 for the treatment of external and perianal warts. Imiquimod upregulates tumor necrosis factor-α, interferon-α, -β, and -γ, leading to decreased HPV DNA/mRNA production and regression of warts [53]. A 2004 efficacy study of 5% imiquimod cream showed that 69.7% (23/34) of patients had complete resolution of warts, 26% (9/34) exhibited 50% to 90% clearance, and one patient showed less than 50% clearance. Seventeen percent of patients who had complete resolution had a recurrence of warts within a 6 months period [54]. Another study showed that 30% of patients had complete resolution and 26% had greater than 50% clearance, with most patients achieving clearance between 8 and 12 weeks of treatment [55].

Imiquimod is made in three preparations of 2.5%, 3.75% and 5% creams. It is applied overnight three times a week until full clearance of warts or for a maximum of 16 weeks. The 3.75% cream is the preferred concentration by most clinicians because it has better efficacy than the 2% cream and fewer adverse effects than the 5% cream [56].

The most common side effects of imiquimod include erythema, erosions, burning, and pruritus [55]. More rare side effects include psoriasiform eruptions, mucosal ulcerations, hyperpigmentation, and one case of meningitis-like presentation with full recovery after drug cessation [57,58]. The major barrier for using this treatment is the exorbitant cost, especially since multiple packs/pumps are necessary due to the long treatment duration needed to achieve resolution [17].

### 5.3. Contact Allergen Immunotherapy

Contact allergen immunotherapy has a long history of use in the treatment of vitiligo and warts. Its mechanism of action is not entirely clear but it is known to elicit a non-specific cell-mediated immune response that triggers death of HPV-infected cells [59]. Currently available contact allergens for warts are squaric acid dibutylester (SADBE), dinitrochlorobenzene (DNCB), and diphenylcyclopropenone (DPCP). A recent study demonstrated that CD3+ T cells decreased production of interleukin-4 (IL-4) and increased production of IL-12 after SADBE treatment. In patients with excellent response to therapy, the percentages of CD3+/IL-10+ T, CD3+/IL-12+ T, and CD3+/IL-4+ T cells were increased, decreased and normalized respectively [60].

A retrospective study reported that 85% of patients treated with DPCP had complete resolution of periungual warts [61]. A small study has also shown successful treatment of chronic facial warts with DPCP [62]. An efficacy study showed that 69% of patients treated with SADBE had clearing of all recalcitrant cutaneous warts [63]. There is some evidence of using SADBE in genital warts, as one study showed complete resolution of lesions in eight out of nine patients within 16 weeks of treatment [64]. Finally, a 1981 clinical study reported that 80% of patients treated with DNCB had complete clearance of warts. DNCB was one of the first studied allergens but is currently not used due to better options, such as DPCP and SADBE [65].

DPCP sensitization is achieved by applying 0.1% DPCP solution in acetone onto the arm, which remains occluded for 48 h. Afterwards, DPCP is applied to lesions with increasing concentrations (starting from 0.1%, up to 3%) until an eczematous reaction is seen at the affected area. This process is repeated every week until the warts clear [61]. There have been other studies that utilize a sensitization of 2% DPCP instead of a concentration of 0.1%. There is no evidence showing that sensitizing with one concentration is more efficacious than the other. The treatment regimen for SADBE is initial sensitization with 1% or 2% SADBE in acetone under occlusion, followed by reintroduction of the allergen to lesions with increasing concentrations (0.5% to 5%) applied every 2 to 4 weeks [63]. Side effects include erythema, desquamation, cutaneous edema, pruritus, burning, and pain. SADBE application to the genital area is problematic as it causes irritation, pruritus, and discomfort for the patient [64]. There have been reports of acute contact dermatitis, blisters, and hypopigmentation as well [63].

## 6. Other

### 6.1. Polyphenon E (Sinecatechins)

Polyphenon E is extracted from green tea leaves and is composed of eight catechins [66]. The primary cathechin is epigallocatechin gallate, which activates capsases, regulates Bcl-2 production, triggers release of proinflammatory cytokines (interleukin-1, interferon-c and tumor necrosis factor alpha) and inhibits telomerase. Via these actions, polyphenon E triggers apoptosis, interrupts HPV transcription and promotes the cell-mediated immune response. These effects lead to destruction of clinically and subclinically HPV-infected cells [17,67].

Polyphenon E 15% and 10% ointments are approved in the US and Europe respectively for the treatment of external genital and perianal warts in immunocompetent adults [68]. There is little data showing the efficacy of polyphenon E in treatment of cutaneous warts. In three major randomized, double-blind, placebo-controlled trials, polyphenon 15% ointment demonstrated clearance rates of 54% to 65% with recurrence rates of 5.9% to 12%. Complete clearance rates were higher in women than in men. Compared to other topical therapies, polyphenon E has the lowest recurrence rate. Imiquimod has rates reported up to 19% and podophyllotoxin has rates up to 38% [17]. In two major double-blind trials, polyphenon E 10% demonstrated similar efficacy as the 15% formulation at 16 weeks for complete and partial clearance. Recurrence rates for polyphenon E 10% were less than 9% [68].

The recommended regimen for polyphenon E 15% is application of 0.5 cm length of ointment onto the wart three times daily for a maximum of 16 weeks [69]. Clinical results are first apparent during the third week of treatment but become more apparent during the fourth to sixth weeks [67]. Side effects of polyphenon E are erythema, pruritus, burning, pain, erosion, ulceration, induration and vesiculation [69]. Of note, the package insert of sinecatechins notes that the medication can weaken condoms and diaphragms [66]. Despite a high rate of these side effects (67% of patients), only 2.3% of patients discontinued therapy in studies [69].

### 6.2. Trichloroacetic Acid

Trichloroacetic acid (TCAA) is a keratolytic agent that is considered to be a form of chemical cautery. When applied, it chemically burns, cauterizes, and erodes the skin and mucosa. The destruction of old, infected epidermal cells allows for the growth of new, healthy cells [70]. Bicholoroacetic acid can also be used for wart therapy.

In an observer blind comparative study, 81% (46/57) of patients using TCAA saw complete resolution of their warts, although there was a high percentage (36%) of wart recurrence within a 2 months period post clearance. Furthermore, there was no significant difference in efficacy between cryotherapy and TCAA [71]. A higher concentration of TCAA (80%–90%) is recommended for use as it is more efficacious than a lower dose (30%) [72].

TCAA is indicated for small, moist lesions, but may also be used for anal or vaginal lesions as well [73]. When applying TCAA to affected lesions, application to the surrounding normal skin must be avoided. Treatment should be repeated once every week until clearance of the wart or a maximum of 10 treatments, whichever comes first. TCAA treatment typically clears warts between 6 and 10 weeks [71]. In cases of over-application, patients should wash the treated wart with soap and sodium bicarbonate to prevent destruction of healthy tissue [70]. Adverse effects include local discomfort, ulceration, and scab formation. TCAA is safe in pregnancy due to the low risk for systemic absorption [70].

## 7. Conclusion

Despite extensive research on cutaneous and anogenital warts, a definitive cure for HPV infection does not exist and management is geared towards symptomatic relief. However, choosing a treatment modality can be challenging. Although many treatment modalities do exist, no single therapy works on all warts and the success of most treatments is user-dependent. Oftentimes, a combination of therapies may be required to achieve complete clearance. Further investigation into the comparative efficacy and cost-effectiveness of treatment options would be helpful in guiding treatment choice.
